# Comparison of the left and right ventricular size and systolic function of low-risk fetuses in the third trimester: Which is more dominant?

**DOI:** 10.3389/fcvm.2023.1052178

**Published:** 2023-03-17

**Authors:** Chen Zhu, Man Li, Cheng-Jie Xu, Meng-Juan Ding, Yu Xiong, Rui Liu, Yun-Yun Ren

**Affiliations:** ^1^ Department of Ultrasound, Obstetrics and Gynecology Hospital of Fudan University, Shanghai, China; ^2^ Department of Information Technology, Obstetrics and Gynecology Hospital of Fudan University, Shanghai, China; ^3^ Department of Obstetrics, Obstetrics and Gynecology Hospital of Fudan University, Shanghai, China

**Keywords:** fetal echocardiography, fetal heart, ultrasound, speckle tracking, fetalHQ, ventricular size, systolic function

## Abstract

**Objective:**

To quantify fetal cardiovascular parameters utilizing fetal-specific 2D speckle tracking technique and to explore the differences in size and systolic function of the left and right ventricles in low-risk pregnancy.

**Methods:**

A prospective cohort study was performed in 453 low-risk single fetuses (28^+0^–39^+6^ weeks) to evaluate ventricular size [i.e., end-diastolic length (EDL), end-systolic length (ESL), end-diastolic diameter (ED), end-systolic diameter (ES), end-diastolic area, end-systolic area, end-diastolic volume (EDV), and end-systolic volume (ESV)] and systolic function [i.e., ejection fraction (EF), stroke volume (SV), cardiac output (CO), cardiac output per kilogram (CO/KG), and stroke volume per kilogram (SV/KG)].

**Results:**

This study showed that (1) the reproducibility of the interobserver and intraobserver measurements was good to excellent (ICC 0.626–0.936); (2) with advancing gestation, fetal ventricular size and systolic function increased, whereas right ventricular (RV) EF decreased and left ventricular (LV) EF was not significantly changed; (3) LV length was longer than RV length in diastole (2.24 vs. 1.96 cm, *P* < 0.001) and systole (1.72 vs. 1.52 cm, *P* < 0.001); (4) LV ED-S1 and ES-S1 were shorter than the RV ED-S1 and ES-S1 (12.87 vs. 13.43 mm, *P* < 0.001; 5.09 vs. 5.61 mm, *P* < 0.001); (5) there were no differences between the LV and RV in EDA or EDV; (6) the mean EDV ratio of right-to-left ventricle was 1.076 (95% CI, 1.038–1.114), and the mean ESV ratio was 1.628 (95% CI, 1.555–1.701); (7) the EF, CO and SV of the LV were greater than the RV (EF: 62.69% vs. 46.09%, *P* < 0.001; CO: 167.85 vs. 128.69 ml, *P* < 0.001; SV: 1.18 vs. 0.88 ml, *P* < 0.001); (8) SV and CO increased with ED-S1 and EDL, but EF was not significantly changed.

**Conclusion:**

Low-risk fetal cardiovascular physiology is characterized by a larger RV volume (especially after 32 weeks) and greater LV outputs (EF, CO, SV, SV/KG and CO/KG).

## Introduction

The size and function of the left and right ventricles in normal fetuses are an important basis and reference for cardiac remodeling studies. Nonetheless, the fetal heart cannot be measured directly *in utero*. Thus, echocardiography is crucial for indirectly obtaining fetal ventricular size and function parameters and includes three general methods. (1) For conventional Doppler and tissue Doppler, the assessment of the fetal ventricular diastolic function of the cardiac cycle is performed with conventional Doppler through the flow velocity of atrioventricular valves (1) and with tissue Doppler through the displacement velocity of the atrioventricular annulus (2). (2) For 2D image and speckle tracking, the length and width of the ventricles are measured first, then the Simpson method is used to calculate the ventricular area and volume (3, 4), and finally the systolic and diastolic volumes are calculated to obtain the parameters of vetricular systolic function. (3) For 3D and 4D sonography the STIC and VOCAL techniques was used to obtain ventricular volume data without assuming geometric shapes (5, 6), then the systolic function parameters are further obtained.

However, these methods have limitations. Doppler velocity is angle-dependent and can make it more difficult to obtain correct images and good measurement reproducibility due to the influence of fetal position (1). It is also more difficult to successfully acquire good images using the 3D/4D method; it takes longer to analyze the images to obtain the data; and the results obtained are only close to (3), or possibly better than (5, 6) 2D measurements. In contrast, the use of 2D speckle tracking is more “cost effective”. Previously, analysis of the rapidly beating fetal heart often resulted in biased measurements of fetal cardiac function parameters due to the low frame rate of ultrasound video and the limitations of the 2D speckle tracking software for adults (7).

The novel fetal-specific speckle tracking software (fetalHQ) solves this problem. The fetalHQ is a specialized software for the quantitative analysis of fetal heart. This software tracks myocardial speckle motion, identifies the endocardial boundaries sensitively and accurately, divides the ventricle into 24 segments, and calculates ventricular volumes using the Simpson method, then further obtain ventricular function parameters (3).

The objectives of this study were (1) to describe a reproducible approach to quantify ventricular volume calculations utilizing fetalHQ and (2) to explore the differences in the size and systolic function of the left and right ventricles in low-risk pregnancy at 28–39 weeks.

## Methods

### Study design and participants

**Figure 1:**
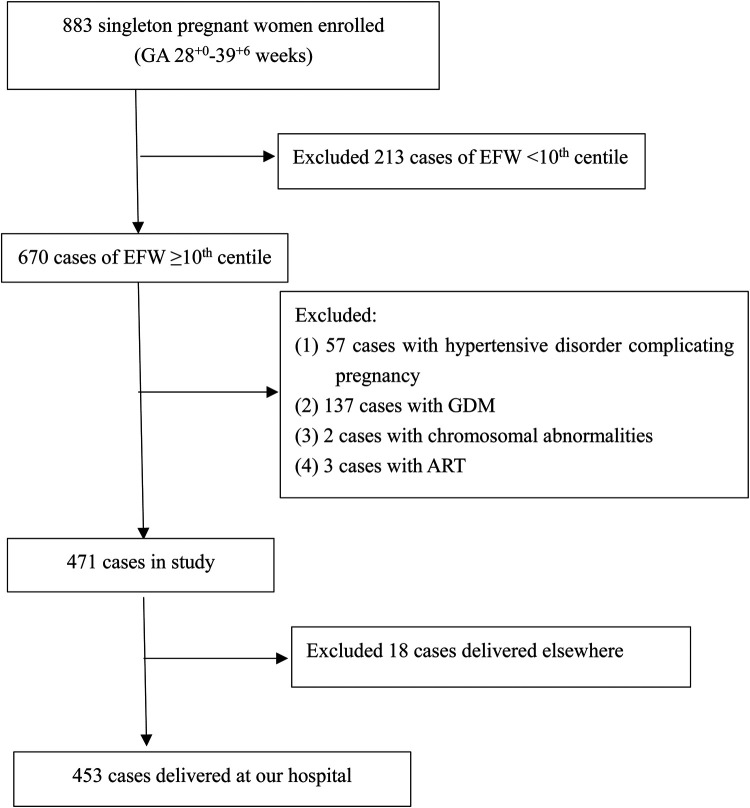
Study flowchart. ART, assisted reproductive technology; GDM, gestational diabetes mellitus.

We conducted a prospective study of singleton pregnant women who received prenatal examinations at the Obstetrics and Gynecology Hospital of Fudan University between April 2020 and July 2021 in Shanghai, China. The flowchart for the selection of the study population is shown in Figure 1. All participants signed a written informed consent form. The study was approved by the Ethics Committee of the Obstetrics and Gynecology Hospital of Fudan University (No. 2020-52).

The inclusion criteria for the study were as follows: (1) singleton pregnancy; (2) gestational age from 28^+0^ to 39^+6^ weeks; (3) complete prenatal ultrasound measurements (including growth ultrasound measurements and fetal echocardiography); and (4) delivery in our hospital with a complete medical history. The exclusion criteria were (1) irregular menstrual cycle, unclear last menstruation, or no crown-lump length record; (2) fetal malformation chromosomal abnormality or structural abnormalities identified by prenatal ultrasound; or (3) any clinical condition potentially associated with cardiovascular remodeling, such as conception by assisted reproductive technology (ART), maternal pregestational diabetes, hypertensive disorder complicating pregnancy (gestational hypertension, chronic hypertension, preeclampsia or eclampsia) or small for gestational age (EFW <10th centile) at the time of the scan.

EFW was calculated with the Hadlock-4 formula (8). According to the updated ISUOG guidelines (9), the EFW percentile standard recommends selecting criteria based on data from prospective low-risk population studies. The INTERGROWTH-21st standard meets this requirement, and the study data included Chinese low-risk fetuses. Therefore, we selected the INTERGROWTH-21st standard (10) as the EFW percentile standard for this study. The neonatal birth weight percentile evaluation criteria used the latest criteria for low-risk neonatal weight in China published in 2021 (11).

### Fetal echocardiography

Two cardiac sonologists with more than 5 years of experience (CZ and ML) performed the ultrasound assessments following a strict protocol (12). All echocardiogram videos were reviewed and approved by the chief sonologist with more than 20 years of experience (Y-YR). All examinations were performed using a Voluson E10 BT19 and BT20 ultrasound device (GE Healthcare, Zipf, Autria) with a transabdominal transducer (GE C2–9, 2–9 MHz, C1–6, 1–6 MHz). All videos were obtained in the absence of fetal body motion and respiratory-like movements, and the pregnant women were asked to hold their breath. Three-second four-chamber loops of the fetal heart were acquired and saved, and during the acquisition time, the heart rate needed to remain stable.

**Figure 2:**
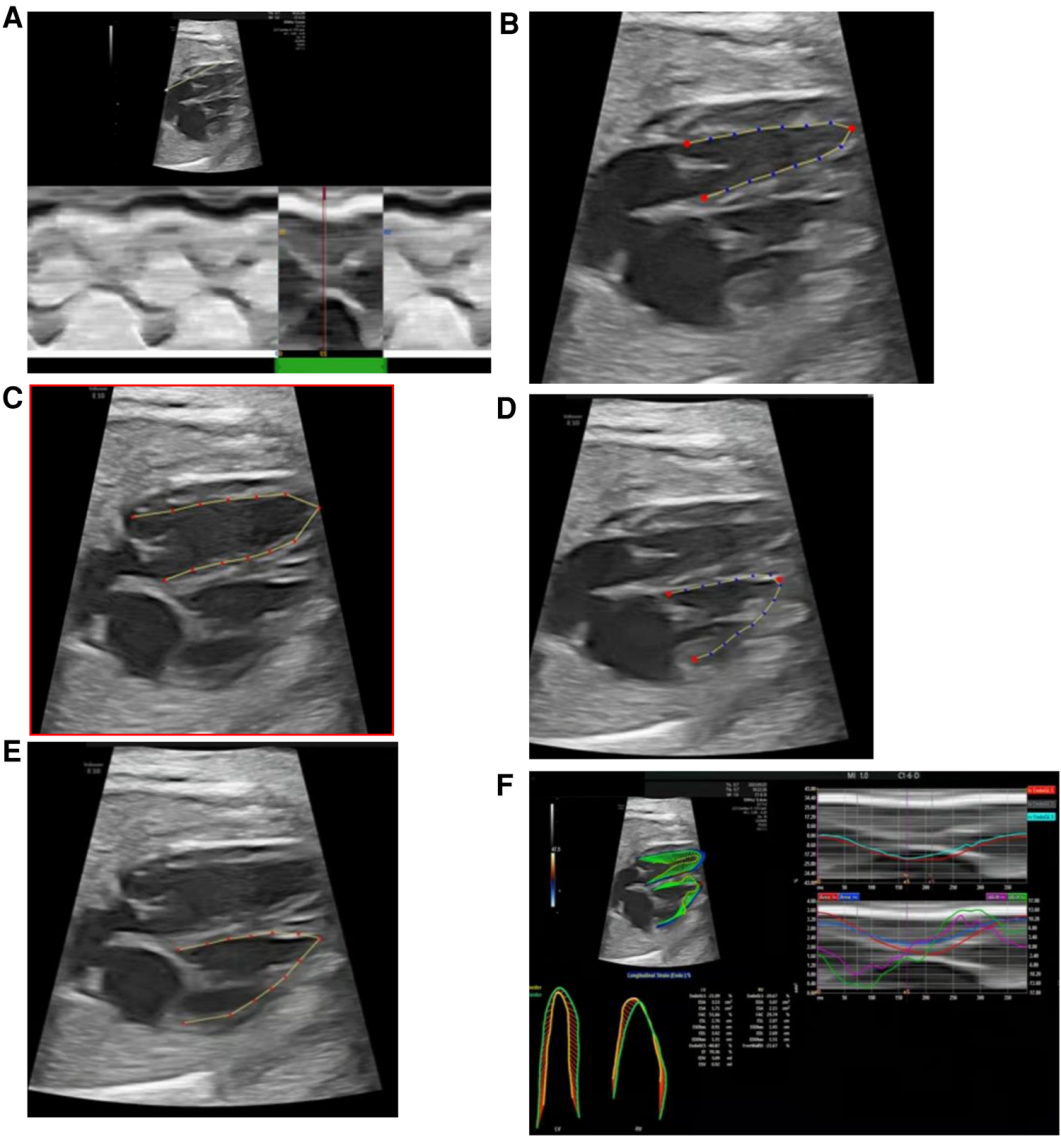
fetalHQ speckle tracking software measures left and right ventricular parameters. (**A**) Selection of one cardiac cycle (M-Mode); (**B**) defining of left ventricular end-systolic endocardial tracing; (**C**) defining of left ventricular end-diastolic tracing; (**D**) defining of right ventricular end-systolic endocardial tracing; (**E**) defining of right ventricular end-diastolic tracing; (**F**) obtaining of results and report.

**Figure 3:**
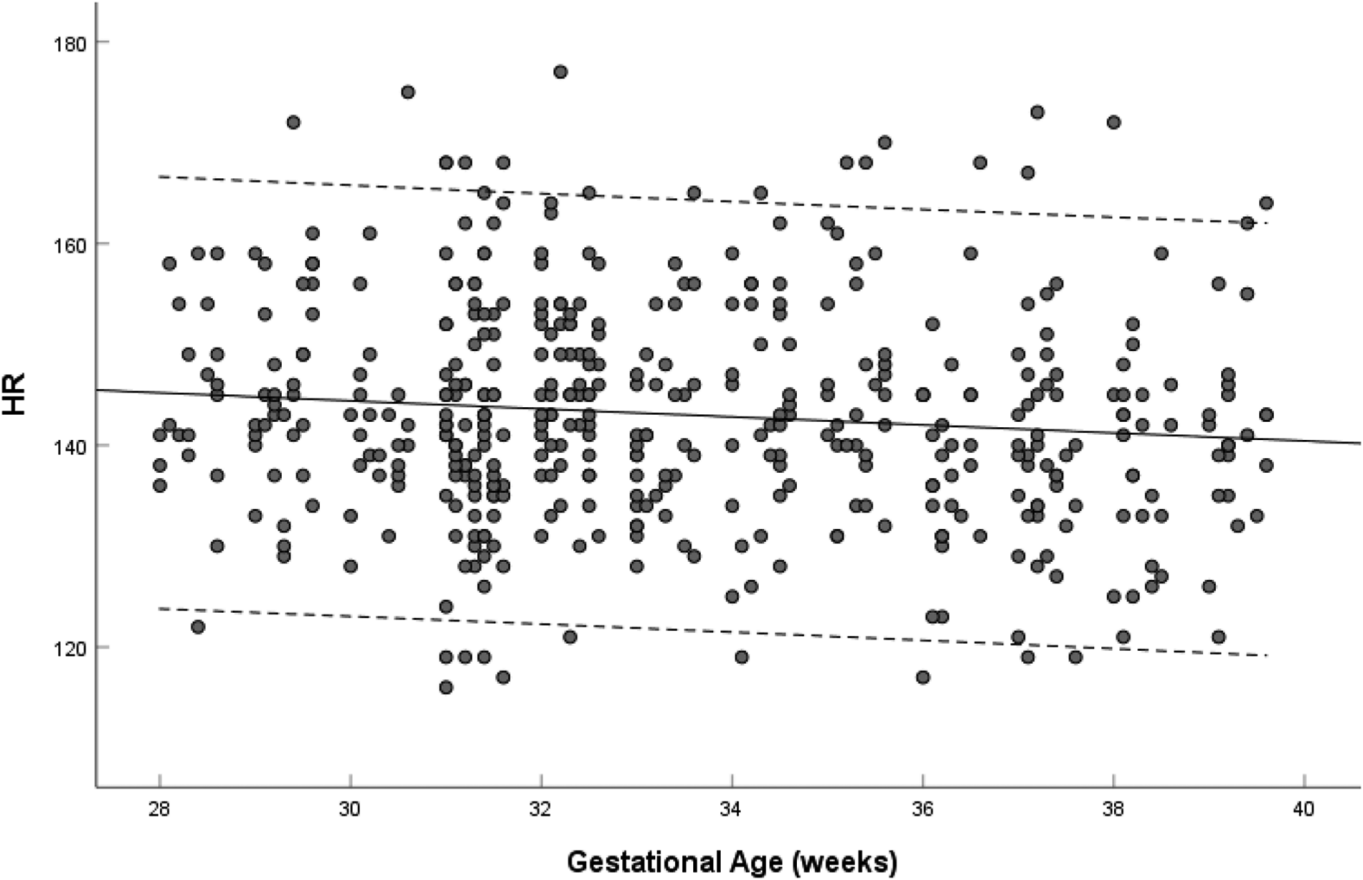
Scatterplots of heart rate, according to gestational age (weeks), in 453 low-risk singleton pregnancies from 28 to 39 weeks gestation. Regression lines with the 5% and 95% confidence intervals are plotted with the regression equation. HR, heart rate.

**Figure 4:**
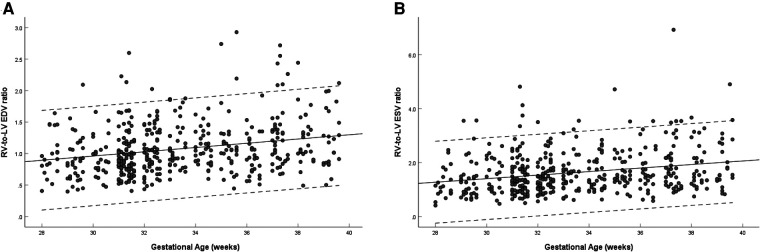
Scatterplots of right-to-left ventricular EDV ratio (**A**) and ESV ratio (**B**), according to gestational age (weeks), in 453 low-risk singleton pregnancies from 28 to 39 weeks gestation. Regression lines with the 5% and 95% confidence intervals are plotted with the regression equation. LV, left ventricle; RV, right ventricle; EDV, end-diastolic volume; ESV, end-systolic volume.

The fetalHQ speckle tracking software (build-in the Voluson E10 ultrasound system) measures ventricular parameters as follows: (1) the M-Mode line is drawn from the apex through the lateral base of the left ventricle (mitral valve lateral anulus), and one cardiac cycle is selected (Figure 2A); (2) left ventricular end-systolic endocardial tracing is defined, and the three red anchor points and blue dots can be adjusted (Figure 2B); (3) left ventricular end-diastolic endocardial tracing is defined, and the red dots can be adjusted (Figure 2C); (4) the endocardial border of the right ventricle at end-systole and end-diastole is traced sequentially (Figures 2D,E); and (5) measurements are completed, with the results exported and reported (Figure 2F).

The parameters of the left and right ventricular size [24-segment end-diastolic transverse diameter (ED), 24-segment end-systolic transverse diameter (ES), end-diastolic length (EDL), end-systolic length (ESL), end-diastolic area (EDA), end-systolic area (ESA), end-diastolic volume (EDV), end-systolic volume (ESV)] and systolic function (EF, SV and CO) were measured and calculated on a four-chamber view by fetalHQ software.

### Reproducibility

The interobserver reproducibility was estimated by comparing the measurements of two ultrasound sonologists (CZ and ML). Both repeatedly practiced tracing data from 30 pregnancies (10 patients per one of the following gestational ages: 28–31, 32–36 or 37–39 weeks) using fetalHQ software, with repeatability tests performed after 1 month. The first test measured the same 30 videos and was completed within 2 days. To assess intraobserver reproducibility, repeated measurements of the stored images of the same 30 videos were taken by the two researchers 1 week later.

### Statistical analysis

Statistical analysis was performed using IBM SPSS Statistics for Windows, Version 25.0 (SPSS Inc., Chicago, IL, United States). Intraclass correlation coefficient (ICC) and 95% confidence interval (CI) were used to determine the interobserver and intraobserver variability of the fetal cardiac measurements. Continuous data that were normally or approximately normally distributed are expressed as the means ± standard deviations (SD), categorical data are expressed as *n* (%), and nonnormal variables are presented as the medians (25th and 75th). Student's *t*-test was used to compare the means, the Mann–Whitney *U* test was used to compare the medians, and the chi-square test or Fisher's exact test was used in the analysis of proportions between the two groups. *P* < 0.05 was considered statistically significant for all comparisons.

## Results

### Population characteristics

Of the 883 singleton pregnancies that were initially eligible for inclusion, 430 were excluded due to FGR noted on fetal ultrasound (*n* = 213), fetal chromosome abnormality (*n* = 2), ART (*n* = 3), maternal hypertension and preeclampsia (*n* = 57), maternal diabetes (*n* = 137) or loss to follow-up (*n* = 18). Therefore, a total of 453 low-risk singleton pregnancies were finally included in the data analysis (Figure 1). Fetal echocardiography data were collected once in each case, and the data from the first scan were selected for those pregnancies with more than two scans. A statistical summary of the characteristics of the research subjects is shown in Table 1. The median maternal age was 30 (28–33) years. Most women (77.9%) were nulliparous. The median gestational age at ultrasound scan was 32.5 (31.2–36.1) weeks, the median gestational age of delivery was 39 (38–40) weeks, the mean birth weight was 3228.5 ± 414.6 g, and the median birth weight percentile was 46.7%, interquartile range (24.0%–75.1%).

**Table 1:** Maternal and pregnancy characteristics of the study population of 453 low-risk singleton pregnancies.

Parameters	Value
Maternal age (years)	30 (28–33)
Pregestational BMI (kg/m^2^)	20.5 (19–22.3)
Prenatal BMI (kg/m^2^)	25.6 (23.7–27.9)
**Parity**	
Nulliparous	353 (77.9)
Parous	100 (22.1)
GA at time of the scan (weeks)	32.5 (31.2–36.1)
EFW	2,028 (1,684–2,518)
EFW centile	52.4 (29.8–75.0)
**Mode of delivery**	
Vaginal	297 (65.6)
Cesarean	156 (34.4)
GA at delivery (weeks)	39 (38–40)
Preterm delivery	15 (3.3)
Birth weight (g)	3226.9 ± 415.2
Birth-weight centile	46.7 (24.0–75.1)
**Neonatal gender**	
Male	217 (47.9)
Female	236 (52.1)
5-min Apgar score*	9 (8–9)*
SGA	38 (8.4)
Adverse perinatal outcome	0
NICU admission	0

*Median (minimum–maximum). Data are given as the mean ± SD, *n* (%), median (interquartile range) GA, gestational age; BMI, body mass index; EFW, estimated fetal weight; NICU, neonatal intensive care unit; SGA, small for gestational age (defined as birth weight <10th centile).

### Reproducibility

The reproducibility analysis showed that after 1 month of training, the interobserver reproducibility was good-to-excellent (ICC 0.626–0.907), and the intraobserver reproducibility was also good-to-excellent (ICC 0.654–0.936) for all of the cardiac parameters evaluated (Table 2).

**Table 2:** Intraclass correlation coefficients and interobserver and intraobserver variability for fetal cardiac measurements.

Parameters	ICC (95% confidence interval)			
	Interobserver		Intraobserver	
	LV	RV	LV	RV
**Ventricular size**				
ED-S1	0.699 (0.458–0.844)	0.691 (0.446–0.84)	0.762 (0.559–0.879)	0.767 (0.566–0.882)
ED-S12	0.704 (0.465–0.847)	0.727 (0.501–0.86)	0.742 (0.526–0.869)	0.792 (0.607–0.895)
ED-S24	0.646 (0.377–0.814)	0.626 (0.348–0.802)	0.871 (0.747–0.937)	0.766 (0.565–0.881)
ES-S1	0.706 (0.469–0.849)	0.759 (0.552–0.877)	0.800 (0.621–0.899)	0.752 (0.542–0.874)
ES-S12	0.695 (0.451–0.842)	0.761 (0.556–0.879)	0.733 (−0.51 to 0.863)	0.776 (0.582–0.887)
ES-S24	0.651 (0.385–0.817)	0.686 (0.438–0.837)	0.736 (0.516–0.865)	0.695 (0.452–0.842)
EDL	0.824 (0.632–0.916)	0.888 (0.740–0.949)	0.824 (0.632–0.916)	0.870 (0.726–0.938)
ESL	0.764 (0.565–0.880)	0.854 (0.638–0.936)	0.846 (0.679–0.926)	0.902 (0.777–0.955)
EDA	0.888 (0.731–0.950)	0.907 (0.803–0.956)	0.918 (0.808–0.963)	0.923 (0.838–0.963)
ESA	0.817 (0.648–0.907)	0.877 (0.683–0.947)	0.857 (0.701–0.932)	0.936 (0.866–0.970)
EDV	0.881 (0.707–0.948)	0.798 (0.619–0.899)	0.926 (0.825–0.967)	0.804 (0.628–0.902)
ESV	0.748 (0.538–0.871)	0.802 (0.624–0.9)	0.842 (0.667–0.925)	0.854 (0.716–0.928)
**Ventricular function**				
EF	0.740 (0.459–0.876)	0.681 (0.429–0.834)	0.712 (0.449–0.856)	0.704 (0.465–0.847)
SV	0.782 (0.541–0.896)	0.668 (0.411–0.827)	0.822 (0.575–0.920)	0.710 (0.475–0.851)
CO	0.803 (0.489–0.915)	0.713 (0.480–0.852)	0.832 (0.620–0.923)	0.742 (0.525–0.868)
SV/KG	0.692 (0.447–0.84)	0.663 (0.402–0.824)	0.732 (0.510–0.863)	0.660 (0.398–0.822)
CO/KG	0.641 (0.370–0.811)	0.626 (0.348–0.802)	0.680 (0.428–0.834)	0.654 (0.390–0.819)

ICC, intraclass correlation coefficient; LV, left ventricle; RV, right ventricle; ED, end-diastolic diameter; ES, end-systolic diameter; S1, segment 1; S12, segment 12; S24, segment 24; EDL, end-diastolic length; ESL, end-systolic length; EDA, end-diastolic area; ESA, end-systolic area; EDV, end-diastolic volume; ESV, end-systolic volume; EF, ejection fraction; SV, stroke volume; CO, cardiac output; KG, kilogram.

### Comparison of left and right ventricular size

Fetal heart rate did not change with gestational age (*R* = 0.019, *P* = 0.679) from 28 to 39 weeks of gestation, median 142 bpm, range (116–177 bpm) (Figure 3).

The left and right ventricular ESL, EDL, ES-S1 and ED-S1 increased with gestational age (left: *R* = 0.420–0.494, *P* < 0.001; right: *R* = 0.0.471–0.581, *P* < 0.001). The EDL and ESL of the left ventricle were longer than those of the right ventricle (EDL: 2.24 ± 0.36 cm vs. 1.96 ± 0.34 cm, *P* < 0.001; ESL: 1.72 ± 0.29 cm vs. 1.52 ± 0.28 cm, *P* < 0.001). The left ventricular ED-S1 and ES-S1 were shorter than the right ventricular ED-S1 and ES-S1 (ED-S1: 12.87 ± 1.78 mm vs. 13.43 ± 2.18 mm, *P* < 0.001; ES-S1: 5.09 ± 0.75 mm vs. 5.61 ± 0.94 mm, *P* < 0.001) (Table 3).

**Table 3:** Comparison of the left and right ventricular size of low-risk fetuses in the third trimester.

Parameters	Left ventricle	Right ventricle	*P* value
**Ventricular size**			
ED Segment 1 (mm)	12.87 ± 1.78	13.43 ± 2.18	<0.001
Segment 12 (mm)	10.82 ± 1.66	11.85 ± 2.05	<0.001
Segment 24 (mm)	2.09 ± 0.44	1.74 ± 0.43	<0.001
ES Segment 1 (mm)	5.09 ± 0.75	5.61 ± 0.94	<0.001
Segment 12 (mm)	3.51 ± 0.76	4.89 ± 0.96	<0.001
Segment 24 (mm)	0.65 ± 0.13	0.73 ± 0.13	<0.001
EDL (cm)	2.24 ± 0.36	1.96 ± 0.34	<0.001
ESL (cm)	1.72 ± 0.29	1.52 ± 0.28	<0.001
EDA (cm^2^)	2.18 ± 0.54	2.11 ± 0.54	0.050
ESA (cm^2^)	1.16 ± 0.34	1.38 ± 0.41	<0.001
EDV (ml)	1.89 ± 0.71	1.92 ± 0.75	0.565
ESV (ml)	0.71 ± 0.33	1.04 ± 0.47	<0.001
**Ventricular function**			
EF (%)	62.69 ± 8.20	46.09 ± 9.06	<0.001
SV (ml)	1.18 ± 0.46	0.88 ± 0.37	<0.001
CO (ml/min)	167.85 ± 64.30	128.69 ± 54.37	<0.001
SV/KG (ml/kg)	0.57 ± 0.18	0.42 ± 0.14	<0.001
CO/KG (ml/min/kg)	81.27 ± 26.38	61.36 ± 21.13	<0.001

Data are given as the mean ± SD. ED, end-diastolic diameter; ES, end-systolic diameter; EDL, end-diastolic length; ESL, end-systolic length; EDA, end-diastolic area; ESA, end-systolic area; EDV, end-diastolic volume; ESV, end-systolic volume; EF, ejection fraction; FS, fractional shortening; SV, stroke volume; CO, cardiac output; KG, kilogram.

The left and right ventricular ESA and EDA increased with advancing gestational age (left: *R* = 0.412–0.504, *P* < 0.001; right: *R* = 0.665–0.710, *P* < 0.001). The left ventricular EDA was not significantly larger than that of the right (2.18 ± 0.54 cm^2^ vs. 2.11 ± 0.54 cm^2^, *P* = 0.050), and the ESA was shorter than that of the right (1.16 ± 0.34 cm^2^ vs. 1.38 ± 0.41 cm^2^, *P* < 0.001) (Table 3).

The left and right ventricular ESV and EDV increased with gestational age (left: *R* = 0.386–0.486, *P* < 0.001; right: *R* = 0.640–0.678, *P* < 0.001). The right-to-left ventricular volume ratios increased with advancing gestational age (EDV ratio: *R* = 0.248, *P* < 0.001; ESV ratio: *R* = 0.252, *P* < 0.001), the mean EDV ratio was 0.987 (95% CI, 0.940–1.034) at 28–32 weeks, 1.170 (95% CI, 1.112–1.229) at 33–39 weeks, and 1.076 (95% CI, 1.038–1.114) at 28–39 weeks (Figure 4A), and the mean ESV ratio was 1.628 (95% CI, 1.555–1.701) at 28–39 weeks (Figure 4B). The left ventricular EDV was not significantly different from the right, whereas the left ventricular ESV was significantly smaller than that of the right (EDV: 1.89 ± 0.71 ml vs. 1.92 ± 0.75 ml, *P* = 0.565; ESV:0.71 ± 0.33 ml vs. 1.04 ± 0.47 ml, *P* < 0.001) (Table 3).

### Comparison of left and right ventricular function

**Figure 5:**
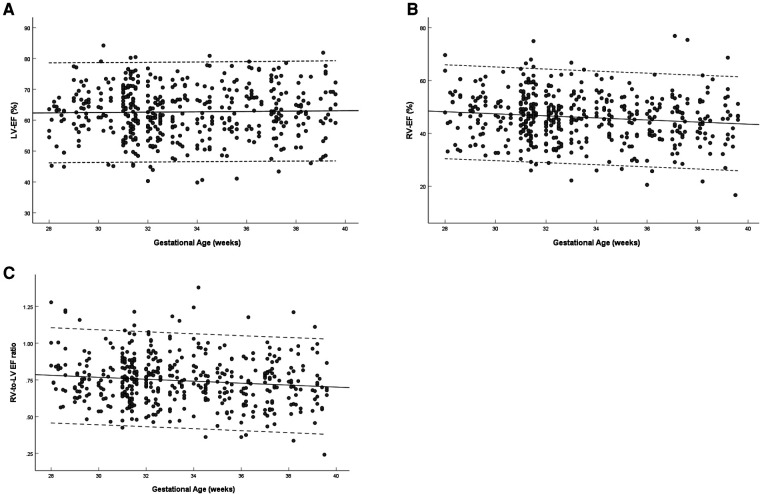
Scatterplots of left ventricular EF (**A**), right ventricular EF (**B**) and right-to-left ventricular EF ratio (**C**), according to gestational age (weeks), in 453 low-risk singleton pregnancies from 28 to 39 weeks gestation. Regression lines with the 5% and 95% confidence intervals are plotted with the regression equation. LV, left ventricle; RV, right ventricle; EF, ejection fraction.

The left ventricular EF showed no significant change with gestational age (*R* = 0.021, *P* = 0.655) (Figure 5A), and the right ventricular EF decreased with gestational age (*R* = −0.131, *P* = 0.005) (Figure 5B). The ratio of right-to-left ventricular EF decreased with gestational age (*R* = −0.123, *P* = 0.009), the mean EF ratio was 0.745 (95% CI, 0.730–0.760) at 28–39 weeks (Figure 5C). The EF of the left ventricle was larger than that of the right ventricle (62.69 ± 8.20% vs. 46.09 ± 9.06%, *P* < 0.001) (Table 3).

**Figure 6:**
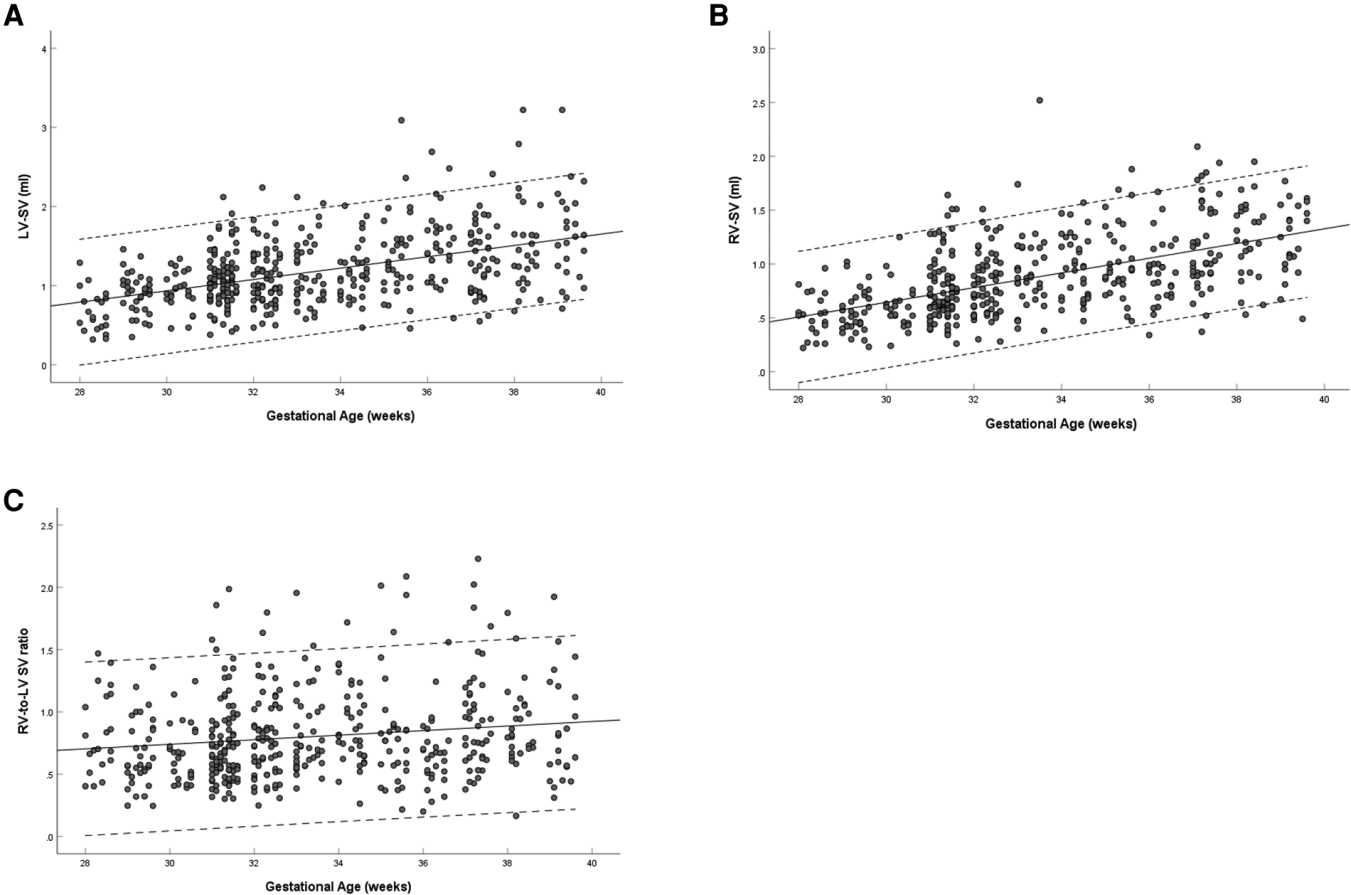
Scatterplots of left ventricular SV (**A**), right ventricular SV (**B**), and right-to-left ventricular SV ratio (**C**), according to gestational age (weeks), in 453 low-risk singleton pregnancies from 28 to 39 weeks gestation. Regression lines with the 5% and 95% confidence intervals are plotted with the regression equation. LV, left ventricle; RV, right ventricle; SV, stroke volume.

The left and right ventricular SV increased with gestational age (left: *R* = 0.477, *P* < 0.001; right: *R* = 0.559, *P* < 0.001) (Figures 6A,B). The ratio of right-to-left ventricular SV decreased with gestational age (*R* = 0.156, *P* = 0.001), the mean SV ratio was 0.802 (95% CI, 0.769–0.835) at 28–39 weeks (Figure 6C). The SV of the left ventricle was larger than that of the right ventricle (1.18 ± 0.46 vs. 0.88 ± 0.37, *P* < 0.001) (Table 3).

**Figure 7:**
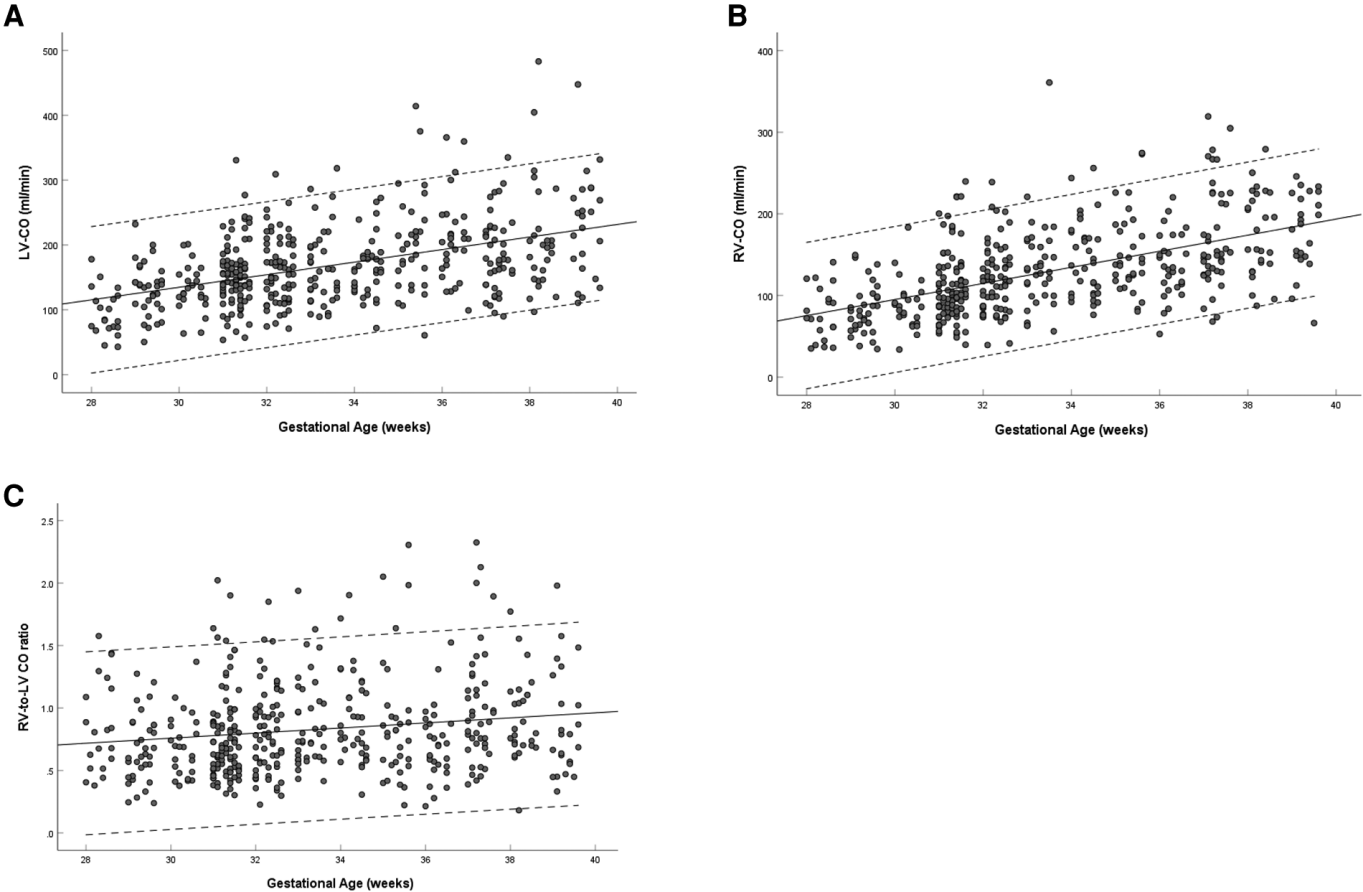
Scatterplots of left ventricular CO (**A**), right ventricular CO (**B**), and right-to-left ventricular CO ratio (**C**), according to gestational age (weeks), in 453 low-risk singleton pregnancies from 28 to 39 weeks gestation. Regression lines with the 5% and 95% confidence intervals are plotted with the regression equation. LV, left ventricle; RV, right ventricle; CO, cardiac output.

The left and right ventricular CO increased with gestational age (left: *R* = 0.458, *P* < 0.001; right: *R* = 0.552, *P* < 0.001) (Figures 7A,B). The ratio of right-to-left ventricular CO decreased with gestational age (*R* = 0.165, *P* < 0.001), the mean CO ratio was 0.828 (95% CI, 0.793–0.863) at 28–39 weeks (Figure 7C). The CO of the left ventricle was larger than that of the right ventricle (167.85 ± 64.30 vs. 128.69 ± 54.37, *P* < 0.001) (Table 3).

The left and right ventricular SV/KG and CO/KG increased with gestational age (left: *R* = 0.209–0.236, *P* < 0.001; right: *R* = 0.011–0.018, *P* = 0.706–0.820). The SV/KG of the left ventricle was larger than that of the right ventricle (0.57 ± 0.18 vs. 0.42 ± 0.14, *P* < 0.001). The CO/KG of the left ventricle was larger than that of the right ventricle (81.27 ± 26.38 vs. 61.36 ± 21.13, *P* < 0.001) (Table 3).

### Relationship between ventricular end-diastolic size and systolic function

The EF of the left and right ventricles did not significantly change with increasing ED-S1 (left: *R* = 0.002, *P* = 0.958; right: *R* = 0.063, *P* = 0.180) or EDL (left: *R* = 0.018, *P* = 0.702; right: *R* = 0.004, *P* = 0.943).

The SV of the left and right ventricles increased with ED-S1 (left: *R* = 0.705, *P* < 0.001; right: *R* = 0.727, *P* < 0.001) and EDL (left: *R* = 0.644, *P* < 0.001; right: *R* = 0.565, *P* < 0.001).

**Figure 8:**
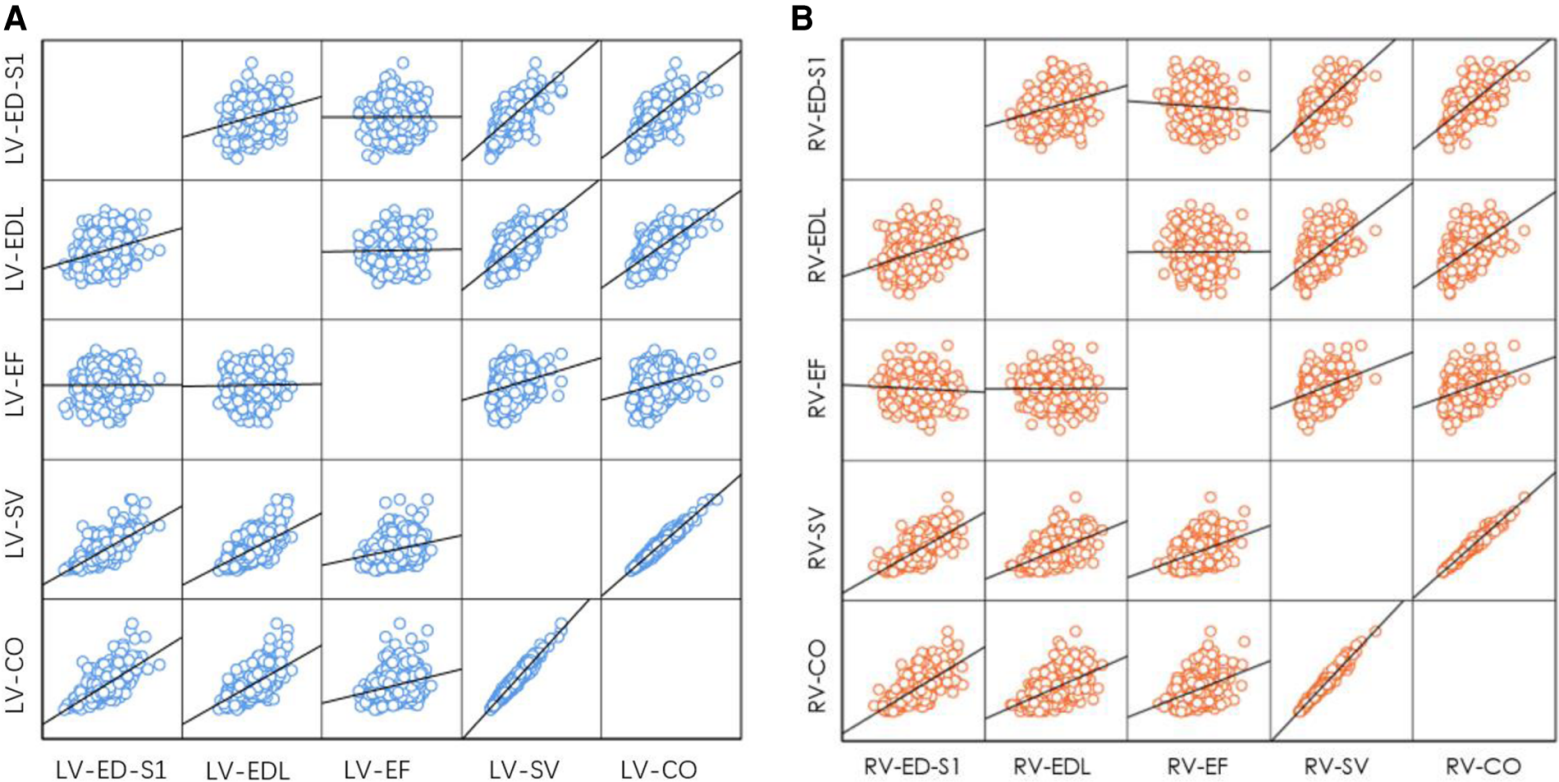
Correlation between end-diastolic ventricular size (ED-S1, EDL) and ventricular systolic function parameters (EF, SV, CO). (**A**) Left ventricle; (**B**) right ventricle. LV, left ventricle; RV, right ventricle; ED-S1, end-diastolic diameter segment 1; EDL, end-diastolic length; EF, ejection fraction; SV, stroke volume; CO, cardiac output.

The CO of the left and right ventricles increased with ED-S1 (left: *R* = 0.694, *P* < 0.001; right: *R* = 0.710, *P* < 0.001) and EDL (left: *R* = 0.630, *P* < 0.001; right: *R* = 0.554, *P* < 0.001) (left: Figure 8A; right: Figure 8B).

## Discussion

The main findings of this study were as follows: (1) the reproducibility of the interobserver and intraobserver measurements was good to excellent after full training; (2) fetal ventricular size and systolic function increased with advancing gestation, whereas right ventricular EF decreased and left ventricular EF was not significantly changed; (3) left ventricular length was longer than the right; (4) left ventricular ED-S1 and ES-S1 were shorter than the right; (5) there were no differences between the left and right ventricles for EDA or EDV; (6) the ratio of right-to-left ventricular volume increased with gestational age, and the right ventricle was found to be volumetrically greater in both EDV and ESV, especially after 32 weeks; (7) The mean EF, CO and SV ratio of right-to-left ventricle were less than 1 at 28–39 weeks, and the left ventricular EF, CO and SV were greater than the right; and (8) SV and CO increased with ED-S1 and EDL, whereas EF was not significantly changed.

### Ventricular dominance in low-risk fetuses in terms of size

In this study, the two ventricular sizes increased with gestational age, whereas the right ventricle size was more associated with gestational age than the left ventricle size. The EDL and ESL of the left ventricle were significantly larger than those of the right ventricle (*P* < 0.001), which is consistent with the findings of DeVore et al. (13) (20–40 weeks). Meanwhile, in our study, the ED-S1 and ED-S12 of the left ventricle were significantly shorter than those of the right ventricle, but the ED-S24 was larger than that of the right ventricle (*P* < 0.001). The left ventricular EDA was not significantly larger than the right ventricular ESA (*P* = 0.05), but the ESA was shorter than the right ventricular EDA (*P* < 0.001). These results indicate that the left ventricle is longer than the right ventricle, that the right ventricle is wider than the left ventricle and that the apical portion of the left ventricle is more rounded than the right ventricle, which is consistent with the anatomy of the right and left ventricles.

Previous studies have reported inconsistent results regarding the comparison of the area and diameter of the two ventricles. For example, DeVore et al. (13) (20–40 weeks) suggested that the EDA and ED-S1 of the left ventricle were significantly larger than those of the right ventricle. Schneider et al. (14) (15–39 weeks) suggested that the EDA of the left ventricle was significantly smaller than that of the right ventricle, and Gabbay-Benziv et al. (15) (16–38 weeks) and Sharland et al. (16) (17–41 weeks) suggested that the left ventricular ED-S1 was significantly smaller than that of the right ventricle.

Ventricular volume changes are most closely related to systolic function. This study analyzed the ratio of right-to-left ventricular volume and found the right ventricle was volumetrically greater in both EDV and ESV, especially after 32 weeks. When we compared ventricular volumes in end-systole, the right ventricle remained greater than the left ventricle, but in end-diastole, there was no difference between right and left ventricles. Many previous studies (5, 17–19) have shown that the EDV of the right ventricle was significantly greater than that of the left ventricle. However, the guidelines for performing the “basic” and “extended basic” cardiac scans (20, 21) and the study by Sutton (22) (20 weeks to term) reported that both ventricles of normal fetal hearts appear similar in size, consistent with the results of our study. Meanwhile, our study demonstrated the right ventricular dominance in the larger volume measurements, especially after 32 weeks.

Changes in ventricular size and morphology, particularly ventricular enlargement, are one of the manifestations of cardiac remodeling (23) and are important in the evaluation of intrauterine growth restriction (24). In contrast, fetal anomalies such as Ebstein's malformation, pulmonary valve stenosis and cardiomyopathy (25) can also present with abnormalities in the left or right ventricular area.

### Ventricular dominance in low-risk fetuses in terms of systolic function

The theory of normal fetal right ventricular dominance that most researchers currently accept was first derived from the results of left and right ventricular output in animal studies (26, 27). However, some researchers have challenged this theory with the equal weight of the right and left ventricles in cadaveric fetal specimens (28). Other researchers have argued that it is unreasonable to use the weight of the heart of a stillborn fetus to infer the predominance of the heart of a living fetus (19). However, the debate on the issue of fetal ventricular dominance is ongoing. There has been debate as to whether the right ventricular output is actually greater, the same as (5, 22, 28) (19–38 weeks), or less than that of the left ventricle (29, 30) (fetal lamb).

The EF, CO and SV can reflect ventricular volume changes. In our study, the mean EF, CO and SV ratio of right-to-left ventricle were less than 1 at 28–39 weeks, and the EF, CO, SV, CO/KG and SV/KG of the left ventricle were greater than those of the right ventricle. Although most studies consider right ventricular dominance in the fetal period, there are authoritative studies that have reported the same results as ours. Hamill et al. (5) (19–38 weeks) suggested that the left ventricular EF was significantly higher than the right ventricular EF, but there was no difference in the CO between the two ventricles using 4D-STIC and VOCAL. Meanwhile, early results from some animal tests support the notion that the output of the left ventricle is greater than that of the right ventricle (29, 30). There is support for the idea that humans have a much larger brain size and metabolic requirements than small animals (31). Under this reasoning, it is also reasonable that the left ventricle needs to contract more than the right ventricle to pump more blood to distribute to the developing brain (5). Anatomically, the right ventricular myofibers are thin, the epi-myocardial circumferential fibers are contiguous with the epi-myocardial oblique fibers of the left ventricle, and the subendomyocardial longitudinal fibers of the right ventricle are contiguous with the fibers of the interventricular septum. The myofibers of the left ventricle are thicker and composed of 3 layers of fibers. In addition to having the same subendomyocardial longitudinal fibers as the right ventricle, the left ventricle has an additional layer of circumferentially oriented fibers in the middle, which play a role in determining the range and extent of myocardial deformation (32).

Therefore, it is reasonable that the contractile force of the left ventricle is higher than that of the right ventricle. On the other hand, umbilical artery resistance decreases with gestational age, and fetal pulmonary vascular resistance is higher than systemic vascular resistance before birth (33). This makes the ESV of the left ventricle smaller than that of the right ventricle, and the EF, SV, and CO, among others, are greater for the left ventricle.

However, it is important to be reminded that many studies, as mentioned above, suggested that the volume of the right ventricle in the fetal period was larger than that of the left ventricle, especially in late pregnancy, which was contrary to the results of this study. The main reasons may be as follows. The structure of the right ventricle is relatively complex, including the right ventricular inflow tract, trabecular part and outflow tract. Because the measurements obtained in this study were based on the four-chamber view, the outflow tract would not be included in the right ventricle, so the EDV, ESV and CO parameters of the right ventricle would be underestimated, which may affect the results of this study.

Abnormal changes in fetal heart function, as well as disproportionate left and right ventricular ratios (34), suggest an abnormal fetal status. Devore et al. showed that (3) EF, SV, SV/KG, CO and CO/kg were significantly reduced in fetuses with severe aortic stenosis and severe anemia; SV, SV/KG, CO and CO/KG were significantly increased in fetuses with cardiomyopathy; and SV and SV/KG were not significantly increased in fetuses with growth restriction.

### Relationship between ventricular end-diastolic size and systolic function

The results of our study showed that the ventricular size (end-diastolic length and diameter) was highly correlated with the output parameters (SV and CO) in low-risk fetuses but had a low correlation with EF. The correlation between ED-S1 and ventricular SV and CO was higher than that of EDL. Therefore, the effect of the changes in ED-S1 on systolic function was greater than that of EDL. Recent literature has reported that fetuses with an EFW <10th centile had an increased area of the 4-chamber view and an abnormal size of the ventricles. The proportion of global transverse width increase was the highest (24). In addition, FGR fetuses have wider left and right ventricles than normal fetuses (35).

### Strengths and limitations

The greatest strength of our study is the finding of higher left ventricular systolic function (including EF, SV, CO, SV/KG and CO/KG) than right ventricle in low-risk fetuses using novel fetal-specific 2D speckle tracking software, followed by the right ventricle was found to be volumetrically greater in both EDV and ESV (especially after 32 weeks), then the finding that ED-S1 has a greater impact on systolic function than EDL.

The limitations are that (1) there remains a lack of a gold standard for validation of ventricular size and systolic function calculation methods, but previous study showed that the method of using volumetric measurements to calculate SV and CO is accurate and reliable (36); and (2) although the combined time for a four-chamber view video acquisition and 2D speckle tracking analysis is 4 min at most, it takes time to learn the professional knowledge that is needed and that a certain sample size for operation training is required to skillfully use the fetalHQ software.

## Conclusion

In summary, the findings of this study suggest that right ventricular dominance was demonstrated by volume measurements, especially after 32 weeks, but that left ventricular dominance was observed in systolic function parameters (EF, CO, SV, SV/KG and CO/KG). ED-S1 has a greater impact on systolic function than EDL. These findings can provide some insights for further research demonstration in the field.

## Data Availability

The raw data supporting the conclusions of this article will be made available by the authors, without undue reservation.
